# Impact of University agricultural research and development spillovers on Chinese agribusiness firms’ performance

**DOI:** 10.1371/journal.pone.0296007

**Published:** 2023-12-15

**Authors:** Yi Wang, Danni Yu

**Affiliations:** 1 Department of Economics and Finance, School of Economics and Management, Qilu Normal University, Jinan, China; 2 Department of International Business, School of Economics and Management, Shandong Youth University of Political Science, Jinan, China; 3 Department of Accounting and Finance, School of Business and Economics, Universiti Putra Malaysia, Serdang, Selangor Darul Ehsan, Malaysia; IBS Hyderabad: ICFAI Business School, INDIA

## Abstract

The spillover effect of university-based agricultural research and development (R&D) has been recognized as a crucial factor contributing to the enhanced performance of the agricultural industry. Nonetheless, the psychological impact of organizational inertia on individuals and groups may shape the influence of such spillovers for agribusiness firms. To date, there has been limited exploration of the interplay between university agricultural R&D spillovers and agribusiness firms. Utilizing panel data from Chinese listed agribusiness firms between 2009 and 2019, our empirical investigation reveals a negative short-term relationship between university agricultural R&D spillovers and agribusiness firm performance due to the similarity in knowledge backgrounds. In the short term, organizational inertia, stemming from familiar and parallel knowledge, renders university agricultural R&D spillovers unfavorable to agribusiness firm performance, thereby reducing their value to the enterprises. Conversely, the long-term influence of university agricultural R&D spillovers on agricultural enterprise performance is positive, as organizational inertia dissipates over time. Additionally, our findings indicate that university non-agricultural R&D spillovers serve to positively moderate the relationship between agricultural R&D spillovers and agribusiness firm performance in the short term, while exerting a negative moderating effect in the long term. Lastly, our analysis reveals a negative correlation between the effect of university agricultural R&D spillovers and the geographical distance between agribusiness firms and universities. This suggests that proximity to academic institutions may play a role in shaping the impact of R&D spillovers on agribusiness performance. In summary, our study highlights the complex dynamics between university agricultural R&D spillovers and agribusiness firm performance, revealing both short-term and long-term effects. We also underscore the importance of considering the influence of organizational inertia and the moderating role of non-agricultural R&D spillovers. Understanding these relationships is crucial for informing strategic decisions and fostering innovation within the agricultural industry.

## 1. Introduction

Agribusiness firms, vital for economic and social sustainability, face heightened market competition due to globalization [1, 2]. In response to these commercial challenges, these firms often invest in enhancing their innovation capabilities to ensure sustainable growth [3]. Agricultural knowledge generated by external R&D sources has emerged as a critical factor for innovation and business growth, becoming indispensable to agribusiness firms [4–7].

Consequently, R&D spillovers, essential for acquiring external agricultural knowledge, have garnered increasing attention [8–10]. R&D spillovers refer to the impact of one organization’s R&D activities on others, arising from the inherent characteristics of non-rivalry and non-exclusiveness [11]. R&D spillovers in the agribusiness sector can manifest in various forms, such as technological innovations that enhance crop yield or resilience, process improvements for food manufacturing, or knowledge-based advancements in supply chain management. These spillovers often emerge from patented technologies developed by universities, cutting-edge research findings published in academic journals, or the tacit knowledge that is transferred when employees trained at universities join agribusiness firms. Within the agricultural sector, the public sector predominantly provides agricultural R&D, resulting in a spillover effect that expands the range of available technologies for agribusiness firms [12, 13]. Universities, as crucial contributors to public agricultural R&D activities, play a significant role in generating these spillover effects [10]. In this context, understanding the dynamics of R&D spillovers is imperative for agribusiness firms seeking to leverage external agricultural knowledge for innovation and growth. By examining the role of universities as key players in the public agricultural R&D landscape, this research aims to provide valuable insights into how agribusiness firms can better navigate and capitalize on these spillover effects to enhance their competitive advantage and contribute to broader economic and social sustainability goals.

Despite the acknowledged significance of R&D spillovers in the agricultural sector, there is a lack of clarity on how agribusiness firms can effectively assimilate and leverage this external knowledge. The problem is twofold: firstly, there is an inherent resistance to change within organizations, known as organizational inertia, which can impede the adoption of new technologies and practices from R&D spillovers. Secondly, the literature has not adequately distinguished between the short-term disruptions and long-term benefits that these spillovers can have on firm performance. This gap in the literature presents a problem for agribusiness firms that seek to navigate these dynamics effectively. The adverse effects of R&D spillovers on short-term performance due to organizational inertia are not well-understood, nor are the potential long-term benefits as firms gradually overcome these initial barriers. Furthermore, while the influence of non-agricultural R&D spillovers has been recognized, their moderating role on the relationship between agricultural R&D spillovers and firm performance has not been sufficiently investigated.

Organizational inertia, a concept deeply rooted in organizational theory, refers to the inherent resistance of firms to change and adapt in response to external stimuli. This resistance often stems from established routines, historical precedents, and deeply ingrained cultural norms [14]. While inertia can provide stability and predictability within an organization, it can also act as a barrier to innovation and adaptation, especially in sectors that are rapidly evolving due to research and technological advancements. In the realm of agribusiness, the continuous flow of research and development (R&D) spillovers from universities presents both opportunities and challenges. These spillovers, encompassing new farming techniques, technological innovations, and sustainable practices, have the potential to revolutionize the agricultural landscape. However, the extent to which agribusiness firms can harness the benefits of these spillovers is significantly influenced by their level of organizational inertia. However, organizational inertia inherently implies a resistance to change [15]. Established routines, processes, and mindsets within agribusiness firms can make it challenging to quickly integrate and benefit from new agricultural R&D spillovers. This resistance can lead to delays in adopting potentially beneficial innovations, thus leading to negative effect on firm performance in the short term.

The adverse influence of R&D spillovers on firms is not constant. Enterprise activities such as exploratory learning and organizational forgetting can help reduce organizational inertia, leading to a positive impact on R&D spillovers [16, 17]. However, these activities typically require a considerable amount of time, resulting in a gradual weakening effect on inertia [14]. As such, the long-term influence of university agricultural R&D spillovers on agribusiness firms differs from their short-term impact. Although previous studies have attempted to address the impact of university R&D spillovers from the agricultural industry’s perspective [18, 19], they have seldom explored the significant distinction between the short-term and long-term effects of university agricultural R&D spillovers at the firm level. This research seeks to fill this gap by examining the varying influences of university R&D spillovers on agribusiness firms over time, providing a more nuanced understanding of the role these spillovers play in shaping firm performance and innovation.

Agribusiness firms experience the influence of not only agricultural R&D spillovers from universities, but also those emanating from non-agricultural fields. The impact of such diverse R&D spillovers on agribusiness enterprises remains unimpeded by organizational inertia, given the marked disparities between the firms’ internal knowledge base and the insights generated by non-agricultural R&D [20, 21]. As posited by Gupta and Govindarajan [22] and Monteiro et al. [23], this particular category of R&D spillover fosters knowledge circulation within organizations, thereby enabling them to capitalize on other knowledge domains and enhance overall performance. Consequently, non-agricultural R&D spillovers from universities positively affect agricultural R&D spillovers, moderating their influence on the performance of agribusiness firms. Nevertheless, extant literature offers scant investigation into the moderating role of universities’ non-agricultural R&D spillovers on the nexus between their agricultural R&D spillovers and the performance of agribusiness enterprises.

In order to address these research gaps, we conducted an investigation into the influence of university agricultural R&D spillovers on the performance of agribusiness firms, employing empirical analysis of a unique panel dataset containing information on the locations of universities and agribusiness firms in China. This study offers two notable contributions by distinguishing between the short-term and long-term impacts of university R&D spillovers. Our findings reveal that, while university agricultural R&D spillovers adversely affect the short-term performance of agribusiness firms, they exert a positive influence in the long run as firms require time to surmount the effects of their existing knowledge. Furthermore, our research uncovers two distinct moderating effects of non-agricultural R&D spillovers from universities on the relationship between agricultural R&D spillovers and agribusiness firm performance in both short-term and long-term contexts, aspects previously unexplored in the literature. China presents a unique case due to its rapidly evolving agribusiness sector and significant investment in agricultural R&D. The period between 2009 and 2019 witnessed profound changes in the Chinese agribusiness landscape, shaped by government initiatives and the strategic alignment of universities with industry objectives. This provided a fertile ground for studying the impacts of R&D spillovers in a transitioning economy, where both the immediate and longer-term effects of such spillovers could be distinctly observed.

This study contributes to the literature in a number of ways. First, this study is pioneering in providing empirical evidence of the direct impact of university agricultural R&D spillovers on the performance of agribusiness firms. While the potential influence of academic research on industry performance has been conceptually recognized, the explicit and measurable effects on agribusiness have not been previously quantified. By employing a unique dataset and focusing specifically on agribusiness firms in China, this study substantiates the positive correlation between university R&D spillovers and agribusiness growth, bridging a critical gap in the empirical literature. Second, our research uniquely dissects the temporal effects of R&D spillovers, distinguishing between immediate and long-term impacts on firm performance. This distinction is crucial as it reveals the dual nature of spillovers—how they initially may disrupt due to organizational inertia but eventually lead to benefits as firms adapt and absorb new knowledge. Such an investigation into the short-term disruptive and long-term advantageous effects provides a nuanced understanding of the innovation adoption lifecycle in agribusiness firms. We reveal that while there’s a negative short-term relationship due to organizational inertia and knowledge similarities, the long-term effects are decidedly positive as this inertia fades. Third, our study employs a unique methodological approach to define university agricultural R&D spillovers. Instead of relying on traditional metrics, we computed the number of agricultural universities situated within a 200-kilometer radius of the firms’ official registration addresses. This innovative approach not only offers a fresh perspective but also facilitates a more nuanced and direct analysis of the relationships at play.

The paper is organized as follows: Section 2 presents the theoretical framework and hypotheses. Section 3 outlines the research methodology. Section 4 provides the empirical analysis, and Section 5 discusses the findings and implications. The paper concludes with Section 6, which offers limitations and recommendations for future research.

## 2. Theoretical framework and hypotheses

### 2.1 University R&D spillover and the performance of agribusiness firms

It is generally believed that spillover effects are caused by the non-competitive and non-exclusive nature of R&D knowledge, and they are an important method by which enterprises acquire knowledge from other organizations [11, 24]. As one of the primary providers of public R&D, universities play a significant role for enterprises in obtaining external knowledge [13]. There are diverse pathways for university agricultural R&D to influence agribusiness firms, which is essential for driving innovation and securing a competitive edge. Collaborative research initiatives and partnerships often serve as a primary channel for this knowledge transfer, with firms and universities engaging in joint research projects that yield mutual benefits [25, 26]. The transition of university graduates and researchers into the industry is another critical vector, infusing fresh expertise and state-of-the-art practices into the agribusiness sector [27]. Informal networks and industry events, including conferences and workshops, further facilitate the exchange of ideas and the latest findings in agricultural R&D [28]. Access to new research outputs is also gained through academic publications and patents, which agribusiness firms can utilize to stay abreast of technological advancements [29]. Additionally, universities often extend consultancy and advisory services, translating complex research findings into practical solutions for business challenges [30].

Qin and Du [31] and Fukugawa [32] found that university R&D spillovers impacted enterprises in a specific area. Furthermore, Zhang, et al. [33] examined the impact of university R&D on Chinese manufacturing firms to validate the relationship between university spillovers and corporate performance. Moreover, Woodward et al. [34] demonstrated a link between university spillover and corporate performance using R&D expenditure at universities as the indicator. Ponds et al. [35] confirmed the impact of university R&D spillover on enterprises from the perspective of university-industry collaboration. More importantly, Anousheh et al. [10] show that university R&D spillovers contribute to the changes in agricultural output. Accordingly, it can be concluded that university R&D spillovers are influential to the performance of agribusiness firms to a certain degree. However, the assimilation of this knowledge is not without its barriers; organizational inertia can significantly dampen a firm’s ability to adapt to and adopt new innovations, necessitating deliberate strategies to mitigate these effects and fully capitalize on the potential of university R&D for sustainable growth and innovation.

### 2.2 Agricultural R&D from university and short-term performance of agribusiness firms

While the existence and impacts of university R&D spillovers on various industries have been well-documented, the specific dynamics between these spillovers and agribusiness firms remain an area ripe for exploration. The intricate interplay between external knowledge and internal organizational structures is pivotal in understanding this relationship.

Liao et al. [20] and Lin et al. [21] delve into the concept of organizational inertia, which is rooted in entrenched problem-solving methodologies derived from a firm’s existing knowledge base. Organizational inertia refers to a firm’s resistance to change due to established routines, processes, and structures. When confronted with new agricultural R&D spillovers, agribusiness firms with high inertia might be slow to adapt or even reject these innovations, leading to short-term performance declines. This inertia, in essence, acts as a barrier, making enterprises less receptive to external R&D spillovers. Delving deeper into the psychological dimensions, Godkin and Allcorn [36] highlight that such inertia can foster a defensive mindset within organizations. This defensiveness can manifest in various dysfunctions at both individual and group levels, ultimately casting a shadow on overall corporate performance. Without proactive organizational interventions, this inertia-driven resistance can persist, leading to suboptimal performance outcomes in the short run.

Assink [37] provides further nuance by illustrating how a firm’s historical experiences and accumulated knowledge can, paradoxically, become impediments, especially when it comes to innovation performance. Firms with a history of success in certain practices might be reluctant to deviate from established paths. The introduction of new R&D findings can challenge these established practices, causing firms to hesitate or delay their adoption. Vecchiat [37] echoes this sentiment, suggesting that a firm’s legacy knowledge can sometimes muddle its adaptability to emerging trends and novel challenges. Lin et al. [21] introduce an intriguing dimension to this discussion by positing that the closer the alignment between a firm’s internal knowledge and incoming external knowledge, the more pronounced the adverse effects of inertia become.

Given the inherent parallels between the knowledge generated from university agricultural research and the foundational knowledge within agribusiness firms, it’s plausible to hypothesize a nuanced relationship between the two. From the perspective of organizational inertia, the negative short-term relationship between agricultural R&D spillovers from universities and agribusiness firm performance can be attributed to the inherent resistance to change within established firms. Therefore, in the short term, the influx of agricultural R&D spillovers from universities may clash with the entrenched knowledge structures within agribusiness firms, leading to a potential negative impact on their performance. The first hypothesis is put forth as follows:

**Hypothesis 1:** The university’s agricultural R&D spillovers are negatively related to the performance of agribusiness firms in the short term.

### 2.3 Moderating effect of university non-agricultural R&D spillovers

Agribusiness firms are influenced by a myriad of knowledge sources, including both agricultural and non-agricultural R&D spillovers emanating from universities. While the knowledge derived from agricultural R&D is inherently familiar to these firms, it often leads to a phenomenon known as organizational inertia. This inertia, rooted in the familiarity and reliance on existing knowledge, can impede innovation, making the direct benefits of agricultural R&D spillovers less pronounced in terms of firm performance.

In juxtaposition, non-agricultural R&D spillovers introduce a diverse and novel knowledge spectrum. Given that this knowledge is not entrenched in the firms’ existing knowledge base, it bypasses the barriers of organizational inertia. Non-agricultural R&D often brings fresh perspectives, methodologies, and technologies that can be applied innovatively in the agricultural sector. For instance, advancements in IT or materials science can lead to the development of better farming equipment, precision agriculture tools, or improved storage solutions [38]. Drawing from the insights of Yang and Maxwell [39], there’s an inherent value in diverse knowledge, and such valuable knowledge tends to disseminate more effectively within an organization. This is further echoed by Gupta and Govindarajan [22], who emphasized the fluidity of high-value knowledge within organizational structures. A broader R&D base, encompassing both agricultural and non-agricultural research, provides agribusiness firms with a diversified knowledge pool. This diversification can lead to more robust solutions, combining the best of both worlds. Building on this, Monteiro et al. [23] and Lai et al. [40] posited that the effective flow and assimilation of such valuable knowledge can catalyze innovation. This innovation, in turn, is bolstered by the acquisition of diverse knowledge, enhancing overall firm performance.

In essence, while agricultural R&D provides domain-specific knowledge crucial for agribusiness firms, non-agricultural R&D spillovers offer complementary insights, technologies, and practices that can enhance and positively moderate the impact of agricultural R&D on firm performance. The synergy between the two can lead to holistic solutions that drive superior outcomes. Consequently, it can be inferred that non-agricultural R&D spillovers, by virtue of their novelty and value, can amplify the positive effects of agricultural R&D spillovers on agribusiness firms. This amplification arises from the stimulation of high-value knowledge flows, fostering an environment conducive to innovation and knowledge acquisition, which ultimately drives superior enterprise performance.

**Hypothesis 2**: University non-agricultural R&D spillovers positively moderate the relationship between agricultural R&D spillovers and the performance of agribusiness firms.

### 2.4 The long-term effect of university agricultural R&D

Agribusiness firms, when first exposed to university agricultural R&D spillovers, often exhibit organizational inertia. This inertia can lead to an initial resistance to the integration of new knowledge, resulting in short-term performance setbacks. However, it’s essential to understand that this inertia is not a static phenomenon. Over time, various enterprise activities can influence and reduce the effects of this inertia.

Gnyawali and Park [41] highlighted the role of co-opetition, a strategy where firms both collaborate and compete, in helping companies navigate and overcome the barriers posed by organizational inertia. In a similar vein, Huang et al. [14] emphasized that broadening the scope of the innovation process can counteract the detrimental effects of inertia, fostering a more conducive environment for organizational growth and adaptation. Overtime, the innovations from R&D spillovers can lead to increased yields, better quality products, and more efficient farming practices.

Further insights from Easterby-Smith and Lyles [42] and Huang et al. [17] introduced the concept of organizational forgetting. This process, where firms intentionally or unintentionally let go of certain knowledge or practices, can be instrumental in diminishing the constraints of organizational inertia. However, it’s pivotal to note that such transformative enterprise activities, including organizational forgetting, exploratory learning, and expansive innovation, are not instantaneous. As Huang et al. [17] elucidated, these processes require a significant duration to manifest fully, implying that their inertia-mitigating effects are inherently long-term. A recent study by Su et al. [43] reveals a U-shaped relationship between agribusiness diversification and technological innovation efficiency in China. The findings emphasize the importance for agribusiness managers to adopt a long-term perspective on diversification. This approach counters the risk of "managerial myopia," which might deter them from investing in R&D projects that have uncertain returns in the long run.

In essence, while the initial phase of integrating university agricultural R&D spillovers can present challenges and costs, the long-term benefits in terms of innovation, efficiency, market positioning, and sustainability can significantly outweigh these short-term hurdles, leading to enhanced performance and growth for agribusiness firms. Given the above dynamics, it becomes evident that while the immediate aftermath of university agricultural R&D spillovers might be met with resistance due to inertia, the long-term landscape is markedly different. The enduring positive contributions of these spillovers to the agricultural sector, combined with the time-dependent reduction in organizational inertia within agribusiness firms, suggest a more favorable long-term impact of university agricultural R&D spillovers compared to the short-term.

**Hypothesis 3**: The university agricultural R&D spillovers have positive effects on the performance of agribusiness firms in the long term.

### 2.5 The effect of geographical proximity on university agricultural R&D spillovers

R&D spillover exhibits characteristics of geographic proximity due to the nature of knowledge flow, which is dependent on individual behavior [35, 44]. Consequently, the effect of R&D spillover may diminish as distance increases, subsequently reducing its impact on company performance. Notably, this geographical proximity is also present in university R&D spillovers. Proximity to universities often facilitates easier and more frequent interactions between researchers and agribusiness professionals. As distance increases, the ease of direct communication and collaboration can decrease, leading to potential barriers in effective knowledge transfer.

Lagendijk and Lorentzen [45] study indicates that shorter geographic distances foster enhanced interaction between two organizations, thereby increasing their knowledge-sharing cooperation. Similarly, Antonelli [46] assert that close geographical proximity facilitates face-to-face communication between organizations, potentially promoting enterprise innovation by disseminating tacit knowledge. In summary, while distance is just one factor, it plays a significant role in determining the extent and effectiveness of agricultural R&D spillovers from universities to agribusiness firms. Proximity facilitates better communication, collaboration, resource sharing, and trust-building, all of which are crucial for maximizing the benefits of R&D spillovers. Based on these findings, the following hypothesis is proposed in this study:

**Hypothesis 4:** The effect of university agricultural R&D spillovers is negatively related to the increase in distance between agribusiness firms and universities.

## 3. Methodology

### 3.1 Data collection

This study utilized a unique panel dataset spanning from 2009 to 2019. Firm-level data were sourced from the China Stock Market & Accounting Research (CSMAR) database, while university R&D spillover data were collected using Google Earth. For subsequent analysis, Chinese universities were classified based on their areas of expertise. To evaluate the effects of agricultural R&D spillovers, agricultural universities specializing in the same fields as agribusiness firms were selected. Non-agricultural R&D spillovers were measured by considering science and technology universities, which supply heterogeneous knowledge to agribusiness firms. To mitigate potential bias arising from the 2008 financial crisis and the COVID-19 pandemic, a panel data analysis was conducted on China’s listed agribusiness firms from 2009 to 2019 to examine the impact of university R&D spillovers. Given the extended period required for enterprises to absorb R&D spillovers, all university R&D spillover data in this study were lagged by one year in the regression analysis.

The previous study posits that agribusiness is inherently linked to value chains, which not only end with the product’s arrival at the consumer’s table but also encompass all the value-adding operations required along the way [47, 48]. Therefore, our definition of agribusiness firms includes entities involved in the production, processing, distribution, and retailing of agricultural products and services [43, 49]. The panel dataset for analysis was constructed as follows: (1) Firm years for which the registered address of agribusiness firms could not be obtained were excluded. (2) Firm years with transaction statuses of particular transfer (PT) were removed to avoid potential errors caused by firms’ improvement in listing status through increased discretionary accrual. (3) Firm years with missing data for any variables planned for analysis during the study period were eliminated. Ultimately, the aforementioned criteria yielded a total of 1,230 firm-year observations for 179 agribusiness firms across the 2009–2019 timeframe. All relevant data are included in the Supporting Information files.

### 3.2 Variables

#### 3.2.1 Independent variables

In this study, university agricultural R&D spillovers were treated as an independent variable. Given the variety of channels through which university agricultural R&D spillovers occur, quantifying their effects is challenging. Consequently, following the approach of Hilary and Hui [50], this study measured university agricultural R&D spillovers by selecting the number of agricultural universities within a certain radius of agribusiness firms’ registered addresses. Considering the characteristics of university R&D spillovers and the distribution of Chinese universities, a distance of 200 kilometers was adopted as the appropriate radius [51, 52]. The following methodology was employed to evaluate knowledge spillover effects: First, a list of agricultural enterprises was compiled by adopting the classification criteria set forth by the China Securities Regulatory Commission in 2012 and utilizing the CSMAR database. Subsequently, the registered addresses of these enterprises were identified, and their geographic coordinates (longitude and latitude) were ascertained using Google Earth. Second, a comprehensive list of agricultural universities was curated, drawing upon the official list disseminated by the Ministry of Education of the People’s Republic of China and the Airuishen alumni network. The geographic coordinates of these universities were also determined via Google Earth.

Third, to compute the distance between universities and listed agricultural enterprises from 2009 to 2019, we employed the methodology outlined in Du et al. [51], using the geographic coordinates as follows: (a) The university’s longitude and latitude were represented as longitudeU and latitudeU, respectively, while those of an agribusiness firm were designated as longitudeF and latitudeF. Utilizing these coordinates, the central angle (θ) was determined through Eq (1). (b) Subsequently, the arc length per radian was calculated using Eq (2). (c) The distance between each agricultural university and the registered address of each agribusiness firm was ascertained by applying Eq (3), as this distance corresponds to the length of a minor arc across the Earth’s surface. Ultimately, the variable UARS (university agricultural R&D spillovers) was defined based on the computed number of agricultural universities located within a 200-kilometer radius of the companies’ registered addresses.


cosθ=sin(latitudeU)×sin(latitudeF)+cos(latitudeU)×cos(latitudeF)×cos(longitudeU−longitudeF) (1)

Radin=(40075.04360)×(180π) (2)

Distance=Radin×(π2−arctan(cosθ1−cosθ2)) (3)

#### 3.2.2 Dependent variable

Previous studies have considered Tobin’s Q, ROE (Return on Equity), and ROA (Return on Assets) as key metrics for evaluating corporate performance. However, the agribusiness sector faces considerable uncertainty in market value due to environmental fluctuations [3]. Consequently, Tobin’s Q and ROE, which are closely tied to market value, may not serve as suitable indicators for assessing the performance of agribusiness firms. As a result, the present study employs Return on Assets (ROA) to more accurately and precisely measure the performance of firms within the agribusiness sector.

#### 3.2.3 Moderating variable

In this investigation, non-agricultural research and development (R&D) spillovers serve as the moderating variable. Although agri-business firms may perceive the engineering and technical knowledge acquired from science and technology universities as unfamiliar, it remains essential for their operations. As such, assessing spillovers from non-agricultural R&D at these institutions is the optimal approach. Following the methodology employed by the University-Agribusiness R&D Spillovers (UARS) study, this research quantifies the impact of university non-agricultural R&D spillovers by identifying science and technology universities situated within a 200-kilometer radius of the agribusiness firms. This measure is referred to as UNARS (University Non-Agricultural R&D Spillovers).

#### 3.2.4. Control variables

Leverage (LEV): Leverage refers to the employment of borrowed funds to amplify return on equity. The influence of leverage on corporate performance has been a subject of debate. While Öhman [53] posits that high leverage can negatively impact corporate performance by decreasing R&D investment, increasing leverage can enhance innovation and performance efficiency for companies experiencing low growth rates. In this study, the leverage of listed agricultural companies is measured using the ratio of total liabilities to total assets.

Equity Nature (EN): The effect of equity nature on corporate performance remains inconclusive in previous research. Choi et al. [54] suggest that state-owned enterprises with close government ties can more easily secure financial support, thus bolstering R&D investment and performance. Conversely, Lin [55] contends that non-state-owned enterprises may outperform state-owned counterparts when utilizing long-term bank loans, although this has not been definitively established for state-owned enterprises. Consequently, equity nature is employed as a control variable to analyze the impact of university R&D spillovers on agribusiness firms. The variable value is set at 1 for state-owned enterprises and 0 for others.

Ownership Concentration (OC): Ownership concentration refers to the proportion of shares held by a company’s major shareholder. A higher ownership concentration may enhance corporate governance, promoting innovation efficiency and improving corporate performance [56]. In this study, ownership concentration is calculated as the ratio of shares held by the largest shareholder to the total number of company shares.

GDP Level (GDP): GDP level serves as an indicator of a region’s overall economic performance. Enterprises situated in developed regions tend to exhibit superior performance compared to those in less developed areas. Drawing on prior research, this study utilizes the natural logarithm of the GDP of the province in which a company operates to evaluate the impact of regional growth on performance. The definitions and sources for all variables involved in the study are provided in Table 1.

**Table 1:** Definition of variables and data source.

Variable	Name	Definition	Source
Performance	The Performance of Agribusiness Firms	The return on assets of agribusiness firms in China	CSMAR database
UARS	The University Agricultural R&D Spillover	The university agricultural R&D spillover for agribusiness firms calculated by the number of agricultural universities within 200 kilometers of the companies’ registered addresses	Google Earth, the list of universities issued by the Ministry of Education of the People’s Republic of China and Airuishen alumni network
UNARS	The University Non-agricultural R&D Spillover	The university non-agricultural R&D spillover for an agribusiness firms calculated by the number of science and technology universities within 200 kilometers of the companies’ registered addresses	Google Earth, the list of universities issued by the Ministry of Education of the People’s Republic of China and Airuishen alumni network
LEV	Leverage	The ratio of total liabilities to total assets of the agribusiness firms	CSMAR database
EN	Equity Nature	The equity nature of the agribusiness firms	CSMAR database
OC	Ownership Concentration	The ratio of the number of shares held by the largest holder to the agribusiness firms’ total shares	CSMAR database
GDP	The regional GDP Level	The GDP level of the province where the agribusiness firms are located	CSMAR database

### 3.3 Models

In this investigation, the influence of university R&D spillovers on the performance of agribusiness firms was examined through the employment of fixed-effects models. The foundational model utilized in this study is delineated as follows. The Hausman test outcome leads to the rejection of the null hypothesis, signifying that fixed-effects regression constitutes the favored methodology. For comprehensive review and reference purposes, the baseline results of the random effect regression analysis have also been detailed and included in a table in the Appendix in supporting information files. In this study, all statistical analyses were conducted using Stata 17.


$${Performance}_{i,t}=\beta_{1}{UARS}_{i,t-1}+\beta_{2}Z_{i,t}+Firm\;fixed\;effects+{Time\;fixed\;effects+\varepsilon }_{i.t}$$ (4)

$${Performance}_{i,t}=\beta_{3}{UARS}_{i,t-1}+\beta_{4}{UNARS}_{i,t-1}+\beta_{5}{UARS}_{i,t-1}\times {UNARS}_{i,t-1}+\beta_{6}Z_{i,t}+Firm\;fixed\;effects+{Time\;fixed\;effects+\varepsilon }_{i,t}$$ (5)

In this context, UARS i, t-1, and UNARS i, t-1 denote the university agricultural R&D spillovers and non-agricultural spillovers, respectively, for firm i in the preceding year (t-1). Performance i, t encompasses variables associated with the performance of agribusiness firm i during year t. Concurrently, Z i, t represents the control variables influencing the performance of agribusiness firms, while ε i, t corresponds to the random disturbance terms.

## 4. Empirical results and discussion

### 4.1 Descriptive analysis and correlation analysis

Table 2 presents descriptive statistics for the primary variables examined in this study, utilizing panel data from 2009 to 2019. The average and median values of performance (ROA) are both 0.05, suggesting a normal distribution for ROA. The mean and standard deviation for the number of agricultural universities situated within a 200-kilometer radius of the listed agribusiness firms are 2.07 and 1.62, respectively. However, UNARS exhibits significant variation, with a standard deviation of 11.63. In relation to the other control variables, the mean leverage ratio stands at 0.38, which is deemed acceptable. The average value of EN is 0.37, signifying that 37% of agricultural enterprises are state-owned entities.

**Table 2:** Descriptive statistics.

Variable	N	Mean	Mid	SD	Min	Max
Performance	1230	0.05	0.05	0.10	-1.88	0.53
UARS	1230	2.07	2.00	1.62	0.00	6.00
UNARS	1230	14.75	13.00	11.63	0.00	47.00
LEV	1230	0.38	0.35	0.23	0.01	4.60
EN	1230	0.37	0.00	0.48	0.00	1.00
OC	1230	0.37	0.37	0.15	0.04	0.96
GDP	1230	28.66	28.74	0.81	25.97	30.01

Please check S1 Data for variables descriptions.

Table 3 displays the correlation coefficients for the variables under study. In line with expectations, the performance of agribusiness firms exhibits a negative and significant association with university agricultural R&D spillover (-0.058), while bearing an insignificant relationship with university non-agricultural R&D spillover (0.043). Furthermore, the performance of agribusiness firms is positively correlated with the shareholding ratio of the largest shareholder (OC) and GDP, and negatively associated with the state-owned enterprise dummy variable (EN) and the leverage ratio (LEV). To further evaluate the possibility of multicollinearity among the test and control variables, variance inflation factor (VIF) tests were conducted on the regression models. Table 4 demonstrates that no evidence of multicollinearity was found, with VIF values ranging from 1.03 to 3.23.

**Table 3:** Correlation matrix.

Correlation	Performance	UARS	UNARS	LEV	EN	OC	GDP	
Performance	1							
UARS	-0.058**	1						
UNARS	0.043	0.763***	1					
LEV	-0.543***	-0.011	-0.071**	1				
EN	-0.011***	0.102***	-0.054*	0.086***	1			
OC	0.180***	0.006	0.049*	-0.118***	0.060**	1		
GDP	0.053***	0.193***	0.327***	-0.036	-0.227***	-0.016	1	

t statistics in parentheses

* p < 0.1

** p < 0.05

*** p < 0.01

**Table 4:** Variance inflation factor.

Variable	UARS	UNARS	LEV	EN	OC	GDP	Mean VIF
VIF	2.54	3.23	1.03	1.13	1.03	1.31	1.69

### 4.2 Empirical analysis

#### 4.2.1. The short-term effect of university agricultural R&D spillovers

Prior research has established that university agricultural R&D spillovers are beneficial to the agricultural industry. However, this study unveils a contrasting outcome in the short term from the vantage point of agribusiness firms. Table 5 delves into the impact of university agricultural R&D spillovers on the performance of listed agribusiness firms. Column 1 presents the simple regression between university agricultural R&D spillovers (UARS) and agribusiness firms’ performance. In order to ensure robustness, we conducted a further analysis using the two-step dynamic system-GMM estimator, as recommended by Arellano and Bover (1995) [57] Arellano & Bover (1995) and Blundell and Bond [58]. The results of this analysis are presented in Column 2 of Table 5. The coefficient of Column 1 and Column 2, which is statistically significant at both the 1% and 10% levels, suggests a negative impact from university agricultural R&D spillovers (UARS)." This outcome supports Hypothesis 1, positing that the agricultural R&D efforts emanating from universities do not enhance the performance of agribusiness firms in the short term. In another word. The university’s agricultural R&D spillovers are negatively related to the performance of agribusiness firms in the short term. These findings align with Lin et al. [21], who discovered that organizational inertia adversely affects an organization’s performance.

**Table 5:** Regression results of the short-term effect of university R&D spillover.

	(1)	(2)
	Performance	Performance
UARS	-0.069***	-0.007*
	(-4.63)	(-1.92)
LEV	-0.276***	-0.364***
	(-19.56)	(-2.81)
EN	0.006	(1.44)
	(0.22)	(0.22)
OC	-0.051	0.053
	(-0.88)	(0.58)
GDP	0.068**	0.004
	(2.32)	(0.26)
_cons	-1.379	0.052
	(-1.64)	(0.10)
AR2		0.257
Hansen test		0.719
Firm-effects	Yes	
Time-effects	Yes	
N	1051	1051

t statistics in parentheses; Please check S1 Data for variables descriptions.

* p < 0.1

** p < 0.05

*** p < 0.01

Tables 6 and 7 explores the role of university non-agricultural R&D spillovers in the relationship between agribusiness firms’ performance and agricultural R&D spillovers, as shown in column 1. By incorporating an interaction term into the model, the impact of university non-agricultural R&D spillover is examined. With a coefficient of 0.003 for the interaction term, university non-agricultural R&D spillovers (UNARS) demonstrate a positive moderating effect between agricultural R&D spillovers (UARS) and agribusiness firms’ performance. In accordance with earlier studies, the results reveal that valuable knowledge, specifically non-agricultural knowledge in this instance, fosters the performance of other types of knowledge, such as agricultural knowledge. More precisely, without the organizational inertia engendered by existing knowledge, university non-agricultural R&D spillovers for agribusiness firms can stimulate high-value knowledge within organizations, thereby increasing internal knowledge flow and enabling agricultural knowledge to yield better performance. This finding also supports Hypothesis 2, which asserts that non-agricultural R&D spillovers from universities facilitate enhanced performance of agribusiness firms by influencing agricultural R&D spillovers.

**Table 6:** Regression results of the short-term effect of university R&D spillover.

	(1)	(2)	(3)
	Performance	Performance	Performance
UARS	-0.138***		
	(-5.27)		
UNARS	0.005		
	(1.37)		
Interaction-term	0.003**		
	(2.45)		
UARS (D)		-3.301***	-6.612***
		(-4.63)	(-5.27)
UNARS (D)			1.794
			(1.37)
Interaction-term(D)			47.720**
			(2.45)
LEV	-0.277***	-0.276***	-0.277***
	(-19.72)	(-19.56)	(-19.72)
EN	0.006	0.006	0.006
	(0.22)	(0.22)	(0.22)
OC	-0.061	-0.051	-0.061
	(-1.05)	(-0.88)	(-1.05)
GDP	0.072**	0.068**	0.072**
	(2.46)	(2.32)	(2.46)
_cons	-1.665*	-1.651**	-1.856**
	(-1.85)	(-2.09)	(-2.33)
Firm-effects	Yes	Yes	Yes
Time-effects	Yes	Yes	Yes
N	1051	1051	1051
Adj. R^2^	0.540	0.536	0.540

t statistics in parentheses

* p < 0.1

** p < 0.05

*** p < 0.01

**Table 7:** Regression results of two-period lagged value of university R&D spillover.

	(1)	(2)
	Performance	Performance
UARS _t-2_	-0.148***	-0.274***
	(-8.79)	(-9.22)
UNARS _t-2_		0.002
		(0.57)
Interaction-term _t-2_		0.007***
		(5.26)
LEV	-0.241***	-0.227***
	(-15.40)	(-14.59)
EN	0.017	0.017
	(0.56)	(0.58)
OC	-0.003	0.000
	(-0.04)	(0.00)
GDP	0.058*	0.052
	(1.77)	(1.61)
_cons	-1.261	-0.741
	(-1.45)	(-0.77)
Firm-effects	Yes	Yes
Time-effects	Yes	Yes
N	882	882
Adj. R^2^	0.569	0.586

t statistics in parentheses; Please check S1 Data for variables descriptions.

* p < 0.1

** p < 0.05

*** p < 0.01

In order to assess the robustness of our findings, we estimated the regression models using the density of agricultural universities and the density of science and technology universities as proxies for university agricultural R&D spillovers and non-agricultural R&D spillovers, rather than utilizing the number of agricultural universities and the number of science and technology universities. The newly derived variables UARS (D) and UNARS (D) were computed by dividing the number of a specific type of university located within 200 kilometers of the agribusiness firms by the total number of such universities in China. Dividing by the total number of such universities in China was an attempt to normalize the data, ensuring that the influence of universities is considered relative to their overall presence in the country. This approach provides a standardized metric that allows for comparative analysis across different regions and agribusiness firms. The results presented in Tables 6 and 7 reveal that the estimated coefficients for all key independent variables align with those reported in columns 1.

Moreover, we employed the two-period lagged value of the key independent variables as measures of university agricultural R&D spillovers and non-agricultural R&D spillovers to perform a robustness check. Following Ullah et al. [59] study, lagged values were utilized to address the endogeneity issue stemming from two-way causality. As illustrated in Table 7, the coefficients on UARSt-2 are negative and statistically significant in columns 4 and 5. Additionally, the coefficient on UNARSt-2 is insignificant, while the interaction term’s coefficient is positive and statistically significant. These results suggest that university agricultural R&D spillovers are negatively associated with agribusiness firms’ performance in the short term, and university non-agricultural R&D spillovers positively moderate the relationship between agricultural R&D spillovers and agribusiness firms’ performance. This corroborates our findings.

#### 4.2.2 The long-term effect of university agricultural R&D spillovers

Previous studies have demonstrated that factors such as innovation and organizational forgetting can weaken organizational inertia [14, 17]. Consequently, the adverse impact of organizational inertia on university agricultural R&D spillovers may be affected by other factors over time. However, these factors typically require a relatively extended period to exert a substantial influence on enterprises. As a result, the long-term effects of university agricultural R&D spillovers are likely to differ from their short-term counterparts.

Utilizing three-period lagged data based on our sample size, we investigate the long-term effects of university R&D spillovers on the performance of agribusiness firms. While we acknowledge that the general innovation process in some industries may take more than ten years, the agribusiness sector operates within a unique context. Given the nature of agricultural cycles, innovations and their subsequent spillovers can manifest and impact firm performance within shorter time frames. Table 8 reveals that university agricultural R&D spillovers (UARSt-3) exert a statistically significant long-term effect on agribusiness firms’ performance, displaying a positive sign, which contrasts with the short-term effect. Notably, concerning non-agricultural R&D spillovers, the coefficient of UNARSt-3 becomes significant, reflecting a reduction in the disparity between non-agricultural and agricultural R&D spillovers as organizational inertia diminishes over time. Additionally, the coefficient of the interaction term is statistically significant but negative at a 1% level, implying that university non-agricultural R&D spillovers continue to moderate; however, their influence on the relationship between agricultural R&D spillovers and agribusiness firms’ performance becomes negative over time. The results presented in Table 8 support Hypothesis 3 by demonstrating that university agricultural R&D spillovers have a long-term positive impact on agribusiness firms’ performance.

**Table 8:** Regression results of the long-term effect of university agricultural R&D spillover.

	(1)	(2)
	Performance	Performance
UARS t-3	0.150***	0.220***
	(6.64)	(5.81)
UNARS t-3		0.013***
		(2.68)
Interaction-term t-3		-0.007***
		(-4.36)
LEV	-0.332***	-0.360***
	(-16.86)	(-17.83)
EN	0.012	0.012
	(0.35)	(0.35)
OC	-0.015	-0.032
	(-0.18)	(-0.37)
GDP	0.124***	0.155***
	(3.00)	(3.76)
cons	-3.214***	-5.358***
	(-2.87)	(-4.42)
Firm-effects	Yes	Yes
Time-effects	Yes	Yes
N	722	722
Adj. R2	0.555	0.572

t statistics in parentheses; Please check S1 Data for variables descriptions.

* p < 0.1

** p < 0.05

*** p < 0.01

#### 4.2.3 The impact of differences in geographical proximity

The empirical analysis conducted above highlights the negative impact of university agricultural R&D spillovers on agribusiness firms. Furthermore, other factors, such as distance, may influence the effectiveness of R&D spillovers. According to Ponds et al. [35] and Qiu et al. [52], university R&D spillovers tend to weaken as distance increases.

To examine the significance of distance in the relationship between university agricultural R&D spillovers and agricultural enterprise performance, we introduce a new independent variable, UARS300, which represents the number of agricultural universities within 300 kilometers of the agribusiness firms.

As illustrated in Table 9, when using the number of universities within 300 kilometers as the independent variable, the impact on the performance of agribusiness firms is diminished compared to the regression result with the number of universities within 200 kilometers as the independent variable. This empirical evidence suggests that distance attenuates the spillover effect of agricultural R&D from universities. Additionally, as distance increases, non-agricultural R&D spillovers from universities do not seem to moderate the impact of agricultural R&D spillovers on agribusiness firms’ performance. Consequently, both the agricultural R&D spillover effect on agribusiness firms’ performance and the moderating effect of non-agricultural R&D spillovers on the relationship between agricultural R&D spillovers and agribusiness firms’ performance decline as distance expands.

**Table 9:** Regression results of different distance.

	Performance	Performance
UARS	-0.069***	
	(-4.63)	
UARS _300_		-0.026**
		(-2.38)
LEV	-0.276***	-0.277***
	(-19.56)	(-19.44)
EN	0.006	0.008
	(0.22)	(0.31)
OC	-0.051	-0.055
	(-0.88)	(-0.94)
GDP	0.068**	0.075**
	(2.32)	(2.57)
_cons	-1.379	-1.600*
	(-1.64)	(-1.89)
Firm-effects	Yes	Yes
Time-effects	Yes	Yes
N	1051	1051
Adj. R^2^	0.536	0.528

t statistics in parentheses; Please check S1 Data for variables descriptions.

* p < 0.1

** p < 0.05

*** p < 0.01

## 5. Conclusion and recommendations for future research

This research primarily examined the influence of university agricultural R&D spillovers on agribusiness firms, while also investigating the disparities between short-term and long-term impacts. Utilizing a panel data analysis of publicly listed agribusiness firms from 2009 to 2019, the empirical investigation revealed that, in the short term, university agricultural R&D spillovers are inversely associated with the performance of agribusiness firms. However, the long-term effects exhibit a positive relationship. Additionally, the study found that university non-agricultural R&D spillovers have a positive moderating effect on the association between agricultural R&D spillovers and agribusiness firms’ performance in the short term, whereas a negative moderating effect is observed in the long term. Lastly, the research determined that the impact of university agricultural R&D spillovers is inversely correlated with the geographical distance between agribusiness firms and academic institutions.

### 5.1 Theoretical and practical implications

This study bridges theoretical exploration and practical application, revealing how university agricultural R&D spillovers shape agribusiness firm performance in China. It uncovers that organizational inertia is a significant theoretical construct that hampers short-term performance due to resistance to external knowledge [15]. Firms often adhere to established procedures and resist incorporating external knowledge derived from university R&D activities [21, 60]. Practically, this suggests a need for policy makers to encourage firms to actively disrupt established norms and foster innovation, enhancing their absorptive capacity and responsiveness to R&D spillovers. For decision makers, the long-term benefits as inertia diminishes, highlighting the positive effects of university R&D, indicating that persistence in innovation strategies eventually pays off [61, 62]. This long-term gain underscores the need for sustained investment in innovation and the cultivation of core competencies that allow firms to adapt to and capitalize on evolving business conditions, particularly in the post-COVID era.

Agriculture is a crucial component of societal sustainability in China, leading to substantial public investment in agricultural R&D to bolster the industry’s performance [63]. However, the challenge that agribusiness firms face in maximizing the output from agricultural R&D investments, as these often yield disproportionate achievements, suggests a gap that policy makers need to address [64, 65]. Therefore, the study emphasizes that non-agricultural R&D spillovers serve as a catalyst for innovation in the short term but presenting challenges in the long term. This duality suggests that agribusiness firms should adopt a strategic approach to integrating diverse knowledge sources, potentially by hiring talent from varied sectors and engaging in cross-disciplinary collaborations to maintain a balance between agricultural and non-agricultural knowledge inputs. Lastly, the practicality of these findings lies in the recommendation for agribusiness firms to forge stronger alliances with universities. Such partnerships can facilitate more effective knowledge management and ensure that firms are positioned to make the most of public R&D resources.

### 5.2 Study limitations, recommendations and future research

Future research could delve deeper into the mechanisms underlying the impact of university agricultural R&D spillovers on agribusiness firms’ performance. Additionally, researchers and scholars might reveal external factors that influence the relationship between agricultural R&D spillovers and the performance of agribusiness firms, thus providing a more comprehensive understanding of the interplay between these entities. While future research can provide valuable insights into the dynamics between agricultural R&D spillovers and firm performance, it is important to acknowledge that such studies may encounter several limitations. These may include difficulties in quantifying the intangible aspects of knowledge spillovers, challenges in isolating the direct impacts of R&D amid a multitude of interacting factors, and potential limitations in data accessibility, particularly proprietary or competitive information that firms may be unwilling to share.

Additionally, the ever-changing nature of agricultural technologies and fluctuating market conditions can introduce significant variability, making it hard to establish long-term causal relationships. Another limitation is the generalizability of findings, as studies may be context-specific and influenced by regional policies, market structures, and the idiosyncrasies of individual agribusiness ecosystems.

Finally, the rapid advancement and adoption of new agricultural technologies can often surpass the pace of academic research, highlighting the need for continuous and timely updates to studies in this domain. Recognizing this dynamic, it is imperative for future research to not only acknowledge these constraints but also to innovate methodological approaches that remain relevant amid such fast-paced changes. To thoroughly explore the causal dynamics between university R&D spillovers and agribusiness firm performance, future studies could benefit from adopting a diverse array of research designs. Longitudinal studies could provide insights into the temporal aspects of these relationships, case studies could offer in-depth analyses of specific contexts, and experimental designs could potentially ascertain causality with greater precision. Embracing such methodological diversity will be crucial in dissecting the intricate causative threads that weave through the fabric of university-industry collaborations in the agricultural sector.

## Supporting information

S1 Data (ZIP)
