# Supplementary material for: Impact of University agricultural research and development spillovers on Chinese agribusiness firms’ performance
Source: PLoS One. 2023 Dec 15;18(12):e0296007. doi: 10.1371/journal.pone.0296007 (PMC10723717; doi:10.1371/journal.pone.0296007)
Supplement: S1 Data — (ZIP) [file pone.0296007.s001.zip › S1 data/S1 data Appendix.docx]

**Appendix**

**Baseline Regression Result with Random Effect**

|  | **Performance** | **Performance** | **Performance** |
| --- | --- | --- | --- |
| UARS | -0.007*** |  |  |
|  | (-2.79) |  |  |
|  |  |  |  |
| UARS(D) |  | -0.358*** |  |
|  |  | (-2.79) |  |
|  |  |  |  |
| UARS t-2 |  |  | -0.010*** |
|  |  |  | (-3.63) |
|  |  |  |  |
| LEV | -0.261*** | -0.261*** | -0.259*** |
|  | (-21.26) | (-21.26) | (-19.62) |
|  |  |  |  |
| EN | 0.011 | 0.011 | 0.015 |
|  | (1.27) | (1.27) | (1.63) |
|  |  |  |  |
| OC | 0.055** | 0.055** | 0.076** |
|  | (2.04) | (2.04) | (2.56) |
|  |  |  |  |
| GDP | 0.011** | 0.011** | 0.015*** |
|  | (2.11) | (2.11) | (2.69) |
|  |  |  |  |
| _cons | -0.175 | -0.175 | -0.305* |
|  | (-1.17) | (-1.17) | (-1.87) |
| N | 1051 | 1051 | 882 |
| R^2^ | 0.33 | 0.33 | 0.36 |

t statistics in parentheses

* p < 0.1, ** p < 0.05, *** p < 0.01
